# Preferences for pre-exposure prophylaxis delivery among HIV-negative pregnant and breastfeeding women in Zambia: evidence from a discrete choice experiment

**DOI:** 10.3389/frph.2024.1350661

**Published:** 2024-10-29

**Authors:** Twaambo Euphemia Hamoonga, Wilbroad Mutale, Jude Igumbor, Samuel Bosomprah, Olujide Arije, Benjamin H. Chi

**Affiliations:** ^1^ Department of Population Studies and Global Health, School of Public Health, University of Zambia, Lusaka, Zambia; ^2^ Department of Public Health, School of Public Health, Faculty of Health Sciences, University of the Witwatersrand, Johannesburg, South Africa; ^3^ Department of Health Systems Management and Policy, School of Public Health, University of Zambia, Lusaka, Zambia; ^4^ Research Department, Centre for Infectious Disease Research in Zambia, Lusaka, Zambia; ^5^ Department of Biostatistics, School of Public Health, University of Ghana, Accra, Ghana; ^6^ Institute of Public Health, Obafemi Awolowo University, Ile-Ife, Nigeria; ^7^ Department of Obstetrics and Gynecology, School of Medicine, University of North Carolina at Chapel Hill, Chapel Hill, NC, United States

**Keywords:** PrEP, HIV/AIDS, discrete choice experiment, pregnant, breastfeeding, Zambia, preferences

## Abstract

**Introduction:**

Pregnant and breastfeeding women at substantial risk for HIV infection in sub-Saharan Africa can benefit from biomedical interventions such as pre-exposure prophylaxis (PrEP). We estimated the benefit that pregnant and breastfeeding women may derive from PrEP service delivery in order to guide PrEP roll-out in the target population in Zambia.

**Methods:**

Between September and December 2021, we conducted a discrete choice experiment (DCE) among a convenient sample of 389 pregnant and breastfeeding women not living with HIV in Lusaka, Zambia. Women aged 18 years or older, with a documented negative HIV result in their antenatal card responded to a structured questionnaire containing 12 choice sets on service delivery attributes of PrEP: waiting time at the facility, travel time to the facility dispensing PrEP, location for PrEP pick-up, health care provider attitude and PrEP supply at each refill. Mixed logit regression analysis was used to determine the participant's willingness to trade off one attribute of PrEP for the other at a 5% significance level. Willingness to wait (WTW) was used to determine the relative utility derived from each attribute against waiting time.

**Results:**

Waiting time at the facility, travel time to the facility, health care provider attitude and amount of PrEP supply at each refill were important attributes of PrEP service delivery (all *p* < 0.01). Participants preferred less waiting time at the facility (*β* = −0.27, *p* < 0.01). Women demonstrated a strong preference for a 3-months’ supply of PrEP (*β* = 1.69, *p* < 0.01). They were willing to wait for 5 h at the facility, walk for more than an hour to a facility dispensing PrEP, encounter a health care provider with a negative attitude in order to receive PrEP enough for 3 months.

**Conclusion:**

Patient-centered approaches can help to inform the design and implementation of PrEP services among pregnant and breastfeeding women. In this study, we found that a reduction in clinic visits—including through multi-month dispensing of PrEP—could improve uptake of services in antenatal and postnatal settings.

## Introduction

Pregnant and breastfeeding women are disproportionately affected by HIV in sub-Saharan Africa (1), and HIV infection during pregnancy has implications for both maternal and child health (2, 3). In order to end HIV as a public health threat, the adoption of safe and effective HIV prevention methods, such as PrEP, are urgently needed (4). In South Africa, for instance, a study showed that offering PrEP to pregnant and breastfeeding women could significantly reduce new HIV infections in the country by 2.5%–7.2% between 2020 and 2030 (5). Another modeling study found that the introduction of PrEP, in combination with other modalities, could reduce HIV incidence by as much as 45% among pregnant and breastfeeding women (6). As a female-controlled HIV prevention technology, PrEP provides additional protection when women fail to negotiate condom use with their partners (7). PrEP provision in antenatal settings, can also lead to reduced mother-to-child transmission of HIV—from *in utero* through breastfeeding (8).

PrEP was first introduced in Zambia in 2016 as a component of HIV prevention services in line with the World Health Organization guidelines at the time. National campaigns have been implemented in the years that followed—including Zambia Ending AIDS (9) with a goal of educating the public about PrEP and generating new demand for the intervention. In 2020, the Zambian Ministry of Health specifically recommended provision of PrEP to pregnant and breastfeeding women at substantial risk for HIV infection (10). Despite the positive attitude and favourable intention to use PrEP among pregnant and breastfeeding women (11), there is evidence that actual use may be low. For example, a study conducted among 658 women not living with HIV seeking effective contraception postpartum showed PrEP uptake at approximately 1% (12). Continuation on PrEP equally remains a challenge among pregnant and breastfeeding women who initiate PrEP (13, 14). Some of the barriers to uptake of and persistence on PrEP could be addressed through differentiated PrEP service delivery, which is a client-centered approach that simplifies and adapts PrEP service provision in ways that serve the needs of people and communities at substantial risk of acquiring HIV (15).

Discrete choice experiments (DCEs) elicit preferences for health care service delivery and can be used to optimize health services. It is a quantitative technique that is grounded in the economic theory of utility maximization and can be useful in eliciting key modifiable attributes among potential users (16, 17), including aspects of decision making related to health outcomes and health care services (18). In the context of HIV care delivery, most studies have focused on understanding preferences for service delivery among people on ART (19, 20). With respect to preferences for PrEP, a recent systematic review found that most studies focused on preferences among men who have sex with men, female sex workers, youth or adolescents, and people who inject drugs (21). Little evidence exists to guide program implementers on how best to deliver PrEP to pregnant and breastfeeding women. To inform PrEP roll-out in antenatal and postnatal settings, we conducted a study to estimate the (1) relative importance of different attributes of PrEP service delivery; (2) trade-offs between the attributes of service delivery, and (3) total satisfaction or benefit respondents derive from PrEP service delivery in antenatal and postnatal settings.

## Methods and materials

### Study design and participants

We conducted a DCE study to quantify service delivery preferences for PrEP among pregnant and breastfeeding women not living with HIV in Lusaka, Zambia. In order to elicit an initial list of attributes and attribute levels to include in the DCE, we conducted in-depth interviews using a semi-structured interview guide as part of our formative work. These interviews were conducted with 24 women who were either pregnant or breastfeeding. We also reviewed existing literature on PrEP service delivery attributes. Based on this literature review—as well as findings from the formative study, and nominal group technique (*n* = 10) (22) —we selected 5 attributes to study. Two of the attributes had 3 response levels and three had 2 levels (Table 1), which yielded a total of 72 potential combinations of attribute levels in a full factorial design. We selected 12 choice sets using a D-efficiency design. This was done using the “dcreate” command in Stata version 16 (StataCorp LLC, College Station, TX, USA), which uses the modified Fedorov algorithm (23).

**Table 1:** Attributes and levels of PrEP service delivery.

Attributes	Levels
Location	Health facility
	Home
Travel time	Less than 1 hour
	1 hour or more
Waiting time	1 hour
	3 hours
	5 hours
Attitude of health care provider	Negative
	Positive
Supply at each refill	1 month
	2 months
	3 months

We conducted this study at Chipata Level 1 hospital, a government facility that is located in peri-urban Lusaka. This is a high volume facility that has a catchment population of over 100,000 and an antenatal clinic that attends to about 400–450 new ANC attendees each month. Given this information, we were confident that we would be able to recruit the required sample size for the study from this single site. The HIV prevalence among pregnant women attended to at this health facility is approximately 16%, a rate that is similar to the national prevalence for women (24). Study eligibility was restricted to pregnant and breastfeeding women aged 18 years or older with a documented HIV-negative result in their antenatal record. All pregnant and breastfeeding women who could not provide written consent or who could neither speak nor understand any of the three languages in which the questionnaire was translated (English, Nyanja and Bemba) were excluded from the study. We recruited study participants from the Maternal and Child Health clinic using convenience sampling. Probabilistic sampling techniques were deemed impractical, since they would have obstructed the flow of service for the high volume of patients seen at the facility.

### Sample size calculation

To calculate the study's sample size, we used the formula:


$$n=\frac{1000c}{ta},$$

where *n* is number of respondents, *t* is number of tasks per respondent (i.e., 12), *a* is the number of alternatives (i.e., 2) and *c* is the largest product of number of levels of any two attributes (i.e., 3 × 3 = 9), which allows comparison of all two-way interactions (25). We further adjusted the sample size upwards using the 2013/2014 Zambia Demographic and Health Survey (ZDHS) response rate for urban women (95.8%) (24) to account for incomplete responses. Our target sample size was 389 pregnant and breastfeeding women.

### Data collection

Study personnel described the study and obtained written informed consent from eligible candidates who were willing to participate. We then collected information about sociodemographic attributes and PrEP knowledge questions using a structured questionnaire developed by the study team. This was followed by questions on preferences for PrEP service delivery. To ensure that participants understood the choice sets, a study team member described the attributes and attribute levels that participants were going to encounter in the DCE. This was done using a sample DCE question which had one choice set. Participants were later presented with 12 choice sets and were asked to choose between the different scenarios which contained the following service delivery attributes of PrEP: (1) place where PrEP is dispensed; (2) waiting time at the facility; (3) travel time to the facility dispensing PrEP; (4) attitude of health care provider and (5) supply at each refill. An example of the choice sets presented to women in our study is illustrated. In all the 12 tasks, the attributes remained constant while the attribute levels varied. The instrument was pre-tested to evaluate feasibility and clarity of questions. The final version was translated into Nyanja and Bemba (commonly spoken local languages) and independently back-translated to English for verification. Trained study personnel administered the questionnaire using face-to-face interviews via paper forms.

### Statistical analysis

We summarized the background characteristics of participants using frequency (percentage) for categorical variables and median (interquartile range) for continuous variables. The primary outcome or utility was women's preferences for PrEP service delivery. We used McFadden's choice model (26) to fit our utility model, which can be expressed mathematically as follows:

$$u_{i}=X_{i}\beta +{\left(z_{i}A\right)}^{\prime }+\;\epsilon_{i}$$

where $u_{i}$ is the utility for case *i*, β is a px1 vector of alternative-specific regression coefficients, and $A=(\alpha_{1},\ldots ,\alpha_{j})$ is a qxJ matrix of case-specific regression coefficients. The elements of the Jx1 vector $\epsilon_{i}$ are independent type 1 (Gumbel-type) extreme-value random variables with mean γ (the Euler-Mascheroni constant, approximately 0.577) and variance $\pi^{2}/6$. The utility model was estimated using mixed logit regression analysis in Stata 16 (StataCorp LLC, College Station, TX, USA).

We determined the relative importance of the attributes using the significance of the coefficients $\beta_{i}$ and their size whereas willingness to wait was estimated as the rate at which participants give up one unit of waiting time for an increase in other attributes. We determined the benefit derived from alternative ways of providing the service using the estimated utility model for different scenarios of attribute levels.

### Ethical approval

The study received approval from the University of Zambia Biomedical Research Ethics Committee in Lusaka, Zambia (934–2020) and the Human Research Ethics Committee at the University of the Witwatersrand in Johannesburg, South Africa (M200564 MED20-02-145). Additional approvals were obtained from the Zambia National Health Research Authority and the Lusaka District Medical Office prior to study activation. Members of the study team went through the information sheet together with study participants in the participants’ preferred language (English, Nyanja or Bemba). After going through the information sheet, participants were given an opportunity to ask questions and seek clarity on any aspects of the study. Participants who agreed to participate in the study were asked to sign consent forms prior to their participation in the study. Those who were unable to provide written consent substituted their signature with their thumb-print. All interviews were conducted in a private place, and participants were assured that their information would be kept confidential. Participants were given a snack in form of a drink and biscuit and transport reimbursement at the end of the interview.

## Results

We conducted face-to-face interviews with 389 pregnant and breastfeeding women receiving care at the study site's Maternal and Child Health Clinic between September and December 2021. Table 2 describes the sociodemographic characteristics of study participants and summarizes the women's knowledge about PrEP and their perceived risk for HIV infection. Fifty percent of the participants were aged below 26 years (IQR: 22–30) and the majority were married and cohabiting with a partner. More than half had acquired secondary education and the majority were not in formal employment (67.9%). Most women never used condoms with their regular sexual partner in the 30 days preceding the interview, and very few perceived themselves at high risk of HIV infection.

**Table 2:** Sociodemographic characteristics of pregnant and breastfeeding women (*n* = 389).

Characteristic	*n* (%)
Age in years, median (IQR)	26 (22–30)
Marital status	
Single	44 (11.3)
Married (cohabiting with partner)	327 (84.1)
Married (not cohabiting with partner)	18 (4.6)
Education	
No formal education	22 (5.7)
Primary	130 (33.4)
Secondary	226 (58.1)
Tertiary	11 (2.8)
Employment status	
Not working	269 (69.2)
Employed for wages	27 (6.9)
Self employed	93 (23.9)
Maternal status	
Pregnant	172 (44.2)
Breastfeeding	217 (55.8)
Gestational age (pregnant women only, *n* = 172))	
First trimester	10 (5.8)
Second trimester	70 (40.7)
Third trimester	92 (53.5)
Condom use in past 30 days	
Never	338 (86.9)
Sometimes	36 (9.2)
Always	15 (3.9)
Lifetime sexual partners, median (range)	2 (1–25)
Partner HIV status	
Known	318 (81.8)
Unknown	71 (18.2)
HIV risk perception	
No risk	158 (40.6)
Low risk	102 (26.2)
Moderate risk	80 (20.6)
High risk	49 (12.6)
PrEP protects against HIV (*n* = 141)^a^	
Yes	98 (69.5)
No	43 (30.5)

^a^ Among women who reported being knowledgeable about PrEP prior to the interview.

### Prep delivery preferences

Each patient in the study responded to 12 choice sets; cumulatively, 4,668 choice sets were presented to study participants. Results from our mixed logit regression analysis (Table 3) show that all the attributes of PrEP service delivery, except location (*p* = 0.98), were important considerations for pregnant and breastfeeding women when deciding to use PrEP. These included waiting time at the facility, travel time to the location dispensing PrEP, attitude of health care provider, and PrEP supply at each refill. Dispensing 3-month's supply of PrEP was the most valued attribute of PrEP delivery (*β* = 1.68; *p* < 0.01), followed by positive health care provider attitude (*β* = 0.97; *p* < 0.01). Waiting time at the venue dispensing PrEP (*β* = −0.57, *p* < 0.01) was more important than travel time to venue dispensing PrEP (*β* = −0.27, *p* < 0.01). The negative coefficients for waiting time and travel time to the facility show that participants in our study preferred lesser waiting times and shorter travel time to the venue dispensing PrEP. Travel time to the facility of less than 1 h (*β* = 0.27; *p* < 0.01) was less important compared to the other attributes of PrEP service delivery.

**Table 3:** Attribute's weight in decision making about PrEP uptake during pregnancy and breastfeeding.

Attribute	Coefficient (*β*)	Robust std. error	*p*-value	Odds ratio	Willingness to wait (h)^a^
Location					
Facility	0.00	–	–	1.00	0.00
Community	0.00	0.04	0.955	1.00	0.13
Travel time					
≥1 hour	0.00			1.00	0.00
<1 hour	0.27	0.07	<0.001	1.30	0.60
Time (h)^b^	−0.57	2.00	<0.001	0.56	
Attitude					
Negative	0.00			1.00	0.00
Positive	0.97	0.19	<0.001	2.64	1.96
Supply at refill					
1 month	0.00			1.00	0.00
2 months	0.87	0.16	<0.001	2.38	1.77
3 months	1.68	0.54	<0.001	5.38	3.33

^a^ Estimated by the ratio of each coefficient to time coefficient.

^b^ Measured on a continuous scale.

Participants had 30% increase in the odds of preferring travel time that is less than 1 h compared to travel time of 1 h or more (OR = 1.30, *p* < 0.001). For every one hour in waiting time, there was a 44% reduction in odds of choosing PrEP services (OR = 0.56, *p* < 0.001). Women were 2.6 times as likely to prefer a health care provider with a positive attitude compared to one with a negative attitude. The odds of preferring 3-month's supply of PrEP were 5.4 times higher than that of receiving 1-month's supply, while the odds of preferring 2-month's supply of PrEP were 2.4 times the odds of preferring 1-month supply (Table 3). Pregnant and breastfeeding women in our study were willing to wait for approximately 2 additional hours in order to be attended to by a health care provider with a positive attitude. They were willing to wait even longer hours (approximately 3 additional hours) in order to receive drugs enough to take them 3 months. The amount of additional time that participants were willing to wait in order to receive their preferred attributes of PrEP delivery suggests that 3-month's supply of PrEP was more important to participants than being attended to by a health care provider with a positive attitude. In stratified analysis, preferences for PrEP service delivery were similar between pregnant and breastfeeding women.

### Alternative strategies of delivering PrEP

We simulated alternative strategies of delivering PrEP in order to determine which model would offer the greatest utility to women who choose to initiate PrEP during pregnancy and breastfeeding. Table 4 details the preference utilities and associated marginal time willingness to wait for different scenarios of delivering PrEP to pregnant and breastfeeding women. The base scenario of delivering PrEP is described by waiting time at the facility of approximately 3 h; travel time to the nearest facility dispensing PrEP of 1 h or more; negative health care provider attitude; and 1-month supply of PrEP at each refill. Assuming that we were to move from the base case scenario to an alternative one that changed only one attribute—while keeping all others constant—increasing the amount of supply of PrEP at each refill would provide women the highest utility from using PrEP. From our results, women were willing to wait an additional 12 h (*β* = 3.38) in order to receive PrEP enough for three months.

**Table 4:** Estimated preference utilities and additional time willingness to wait for change in PrEP delivery.

Waiting time	Location	Travel time	Attitude	Supply	Benefit score	Additional time willingness to wait
Base case						
3 h	Health facility	1 h or more	Negative	1 month	–	–
Changing only one attribute of PrEP service delivery						
1 h	–	–	–	–	0.54	2.00
5 h	–	–	–	–	−0.54	−2.00
–	–	Less than 1 h	–	–	0.27	0.93
–	–	–	Positive	–	0.97	3.40
–	–	–	–	2 months	0.87	3.04
–	–	–	–	3 months	3.38	11.78^a^
Changing two attributes of PrEP service delivery						
1 h	–	Less than 1 h	–	–	0.81	2.93
1 h	–	–	Positive	–	1.51	5.4
1 h	–	–	–	2 months	1.41	5.04
1 h	–	–	–	3 months	3.92	13.78
–	–	Less than 1 h	Positive	–	1.24	4.33
–	–	Less than 1 h	–	2 months	1.14	3.97
–	–	Less than 1 h	–	3 months	3.65	12.71
–	–	–	Positive	2 months	1.84	6.44
–	–	–	Positive	3 months	4.35	15.18^a^
5 h	–	Less than 1 h	–	–	−0.27	−1.07
5 h	–	–	Positive	–	0.43	1.40
5 h	–	–	–	2 months	0.33	1.04
5 h	–	–	–	3 months	2.84	9.78
Changing three attributes of PrEP service delivery						
1 h	–	Less than 1 h	Positive	–	1.78	6.33
1 h	–	Less than 1 h	–	2 months	1.68	5.97
1 h	–	Less than 1 h	–	3 months	4.19	14.71
1 h	–	–	Positive	2 months	2.38	8.44
1 h	–	–	Positive	3 months	4.89	17.18^a^
–	–	Less than 1 h	Positive	2 months	2.11	7.37
–	–	Less than 1 h	Positive	3 months	4.62	16.11
5 h	–	Less than 1 h	Positive	–	0.70	2.33
5 h	–	Less than 1 h	–	2 months	0.60	1.97
5 h	–	Less than 1 h	–	3 months	3.11	10.71
5 h	–	–	Positive	2 months	1.30	4.44
5 h	–	–	Positive	3 months	3.81	13.18
Changing four attributes of PrEP service delivery						
1 h	–	Less than 1 h	Positive	2 months	2.65	9.37
1 h	–	Less than 1 h	Positive	3 months	5.16	18.11^a^
5 h	–	Less than 1 h	Positive	2 months	1.57	5.37
5 h	–	Less than 1 h	Positive	3 months	4.08	14.11

^a^ Alternative ways of providing PrEP delivery to pregnant and breastfeeding women yielding the highest benefit score.

We compared alternative scenarios that changed two attributes. Based on our responses, women would derive more value from using PrEP if they received 3-months’ supply of PrEP at each refill and encountered a health care provider with a positive attitude (*β* = 4.35 and additional time willingness to wait of slightly over 15 h). In the event that three attributes of PrEP service delivery were changed, women would benefit more if they received PrEP enough for 3 months, encountered a health care provider with a positive attitude, and waited not more than 1 h at the facility (*β* = 4.89, and additional time willingness to wait of just slightly over 17 h). Changing all the four attributes viewed as important considerations when deciding to use PrEP would yield a utility of 5.16 and women would be willing to wait an additional 18 h for this change in PrEP service delivery. Since the utilities in the scenarios presented above are greater than 0, it means that pregnant and breastfeeding women would benefit from such changes in PrEP delivery.

## Discussion

Our study revealed that waiting time at the facility, travel time to the facility dispensing PrEP, health care provider attitude and amount of PrEP supply at each refill were important attributes of PrEP service delivery among pregnant and breastfeeding women. These findings on preferred attributes of PrEP service delivery are consistent with WHO guidance (27). From our findings, optimizing the way PrEP is currently being delivered would be beneficial to women who choose to use it for HIV prevention during pregnancy and breastfeeding.

In this study, participants demonstrated a relatively strong preference for a 3-month supply of PrEP. In fact, this was the most preferred single attribute likely to influence PrEP uptake among pregnant and breastfeeding women in our study. There are a number of possible explanations. For example, it is possible that women preferred the 3-month supply (as opposed to shorter refill periods) to avoid costs associated with frequent clinic visits for PrEP refills. Multi-month refills can address other key barriers to PrEP uptake such as transport expenses and lost wages arising from long waiting hours at the facility. However, there may be disadvantages to such an approach. Caution should be taken to ensure that multi-month scripting of PrEP is not offered to women who intend to use PrEP for a short duration, who are uncertain of their period of risk, and/or have medical conditions that require more intensive monitoring (28).

Health care provider attitude was the second most preferred single attribute of PrEP service delivery among study participants, a finding similar to other studies in the region. In Kenya and Uganda, for instance, women highlighted the importance of health care providers with respect to PrEP messaging and adherence (29). Poor treatment by staff was cited as a reason for non-adherence among some women in FEM-PrEP in Kenya and South Africa (30). In another PrEP study in South Africa, positive encounters (friendly, patient and respectful) with trial staff promoted participation (31). Similar findings on barriers to PrEP uptake were also reported elsewhere (32). Findings from our qualitative study among pregnant and breastfeeding women were similarly consistent: a negative health care provider attitude can discourage potential users from accessing PrEP for HIV prevention during pregnancy and breastfeeding (22).

Although circumstances surrounding uptake of PrEP may differ from those around HIV treatment, both PrEP and ART are biomedical interventions and lessons learnt from ART studies could inform PrEP delivery. In a DCE that was conducted in Zambia to quantify facility-based preferences for ART services, patients were willing to wait 19 h more to see nice rather than rude providers. Patients were willing to accept a facility located 10 km from home (as opposed to 5) that required 5 h of waiting per visit (as opposed to 1 h) and that dispensed 3 months of medications (instead of 5) in order to access nice (as opposed to rude) providers (19). Similar findings were reported among patients on ART who were lost to follow up in Nigeria, Tanzania and Uganda (33).

Waiting time and travel time to the facility dispensing PrEP were equally important attributes that influenced the likelihood of PrEP uptake among women interviewed in our study. Participants preferred less waiting time and shorter travel time to the facility. These results are supported by several other studies that reported long waiting hours and long travel time to health facilities as health systems barriers to uptake of services. In one study, living far from the clinic created difficulties to visit the clinic while lengthy clinic visits were viewed as extremely disruptive and demotivating, lack of consideration for participants’ time (31). Similar findings were reported in other studies in the region (32, 34).

Our findings have important public health implications. Although waiting time, travel time to the facility, amount of PrEP supply and health care provider attitude all influenced the likelihood of PrEP uptake in our study population, addressing all barriers of service uptake amidst resource constraints may require adopting an incremental approach. This approach would require an understanding of the value derived from each of the preferred attributes of PrEP service delivery. Our study quantifies the trade-offs that women are willing to make in order to derive value from using PrEP during pregnancy and breastfeeding. In view of our findings, prioritizing pregnant and breastfeeding women for multi-month dispensing of PrEP as well as improving client-health care provider relationships, through improved health care provider attitude may have a positive impact on PrEP uptake in antenatal and postnatal settings. Increased uptake of PrEP in the target population could significantly reduce the risk of maternal HIV infections and thereby avert the negative impact of HIV infections on women, including increased risk of maternal morbidity and mortality, reduced fecundity, miscarriages and still births (35, 36), among others. Further, this would contribute towards the attainment of sustainable development goal number 3 on the elimination of HIV mother-to-child transmission.

Effective delivery of HIV prevention to pregnant and breastfeeding women is essential. Women who newly acquire HIV face significant lifetime risk for morbidity and mortality; when this occurs during the antenatal and postnatal periods, there is further concern for onward vertical HIV transmission. PrEP is an important component of comprehensive HIV prevention, but its delivery within maternal-child health platforms can present challenges. Patient-centric approaches—such as differentiated service delivery to PrEP implementation—may hold promise. These strategies respond to client needs and preferences, and can overcome known individual and health system barriers to PrEP uptake and continuation (37). The WHO framework for differentiated service delivery includes four building blocks, including service location (where), frequency (when), package (what), and provider (who) (27). This study was designed to interrogate many of these features and better understand the preferences of an often overlooked population: pregnant and breastfeeding women. Our findings provide insights about the relative weight of these attributes in patient decision-making and, as such, will help to inform future interventions for this population in need.

Despite the strengths of our work, we note several limitations. First, theoretical uptake of PrEP used in our study limits our understanding of preferences for PrEP delivery in real-life settings. At the time of study implementation, PrEP was not yet integrated into maternal and child health platforms, and so study findings were meant to inform programs that would do so. Second, we limited the study to daily oral PrEP, since that was the only formulation available at the time that the study was conducted. Findings on preferences could be different with long-acting formulations such as injectable cabotegravir or the dapivirine vaginal ring; however, at the time of data collection, neither was approved for HIV prevention in Zambia. Third, we limited PrEP service delivery attributes to only five and therefore were unable to measure other attributes that could potentially influence women's decision making process, such as location for pick-up of PrEP within the health facility (e.g., ART department, out-patient department, maternal and child health department). However, presenting more attributes and attribute levels is associated with greater cognitive difficulty of completing a DCE for participants (16). Fourth, our sample was drawn from a single health facility in a peri-urban setting and therefore results may not be extrapolated to women in rural parts of Zambia. Fifth, we did not determine the proportion of women in sero-discordant relationships, and therefore did not fully characterize the risk of HIV faced by participants. Even with these limitations, our study is among the first to investigate preferences for PrEP service delivery among pregnant and breastfeeding populations and will help to inform PrEP scale-up in Zambia and other similar settings.

## Conclusion

Our study shows that waiting time at the facility, travel time to the facility dispensing PrEP, health care provider attitude and supply at each refill are key attributes of service delivery likely to influence uptake of PrEP among eligible pregnant and breastfeeding women. As national programs seek to implement differentiated service delivery for PrEP—especially initiatives for pregnant and breastfeeding women—such efforts to prioritize individual needs and preferences are urgently needed.

## Data Availability

The raw data supporting the conclusions of this article will be made available by the authors, without undue reservation.
